# The legacy effects of keystone individuals on collective behaviour scale to how long they remain within a group

**DOI:** 10.1098/rspb.2015.1766

**Published:** 2015-09-07

**Authors:** Jonathan N. Pruitt, Noa Pinter-Wollman

**Affiliations:** 1 Department of Biological Sciences, University of Pittsburgh, Pittsburgh, PA 15260, USA; 2 BioCircuits Institute, University of California San Diego, La Jolla, CA 92093, USA

**Keywords:** boldness, collective memory, foraging, hysteresis, personality, social dynamics

## Abstract

The collective behaviour of social groups is often strongly influenced by one or few individuals, termed here ‘keystone individuals’. We examined whether the influence of keystone individuals on collective behaviour lingers after their departure and whether these lingering effects scale with their tenure in the group. In the social spider, *Stegodyphus dumicola*, colonies' boldest individuals wield a disproportionately large influence over colony behaviour. We experimentally manipulated keystones' tenure in laboratory-housed colonies and tracked their legacy effects on collective prey capture following their removal. We found that bolder keystones caused more aggressive collective foraging behaviour and catalysed greater inter-individual variation in boldness within their colonies. The longer keystones remained in a colony, the longer both of these effects lingered after their departure. Our data demonstrate that, long after their disappearance, keystones have large and lasting effects on social dynamics at both the individual and colony levels.

## 1. Introduction

The ability to execute effective collective behaviour is vital for social groups. The coordinated gliding of fish schools when evading predators or the emergent nest structures of social insects represent collective adaptations that afford groups advantages that are not achievable for solitary individuals [1]. Such collective traits have captured the imagination of scientists including ecologists [2,3], behaviourists [4,5], mathematicians [6] and engineers [7,8], perhaps, more than anything else, because these collective traits are thought to emerge without central control [9]. In classic models of collective behaviour [10,11], individuals are treated as functionally equivalent. Yet, a cursory glance at any group reveals that, even among clones, no two individuals behave in precisely the same way [12]. Only recently have models of collective behaviour begun to predict how such behavioural variation is expected to impact collective outcomes (e.g. [13,14]). We consider here an extreme case of how individual variation can impact collective behaviour, where the behavioural traits of just one or a few highly influential individuals shape the behaviour of entire societies.

We define individuals that exhibit a disproportionate large influence over collective behaviour as *keystone individuals* (or just ‘keystones’). Though one may reason that keystone individuals might be a relatively rare phenomenon, a recent literature review on the topic identified more than 80 case studies where just one or a few highly influential group members shape group behaviour and success [15]. Movement leaders [16–19], knowledgeable tutors [20–22], hyperaggressive males [23], catalytic individuals [24,25] and disease superspreaders [26] represent just some of the ways in which keystone phenotypes can influence group function.

The reliance of a group on one influential individual may reduce its robustness to perturbation. For instance, groups' ideal collective phenotype could be compromised if their keystone individuals leave or die [27]. Such groups might also be more susceptible to manipulation by predators or pathogens because the keystone may serve as a fulcrum by which an exploitative agent can manipulate the entire group [28,29]. A possible solution to this potential system fragility would be that keystone individuals impose long-lasting changes on the behaviour of other group members which, in turn, could maintain their influence over the group's collective phenotypes even after their departure. Although catalytic effects by keystone individuals have been suggested for some social systems [24,25,30], there are few experimentally verified examples of these effects being long lasting (but see: [31,32]), and even fewer studies have determined what factors control the duration of these effects. Determining what controls the onset and duration of behavioural changes induced by keystone individuals is important for understanding the robustness of collective systems.

Social spiders of the genus *Stegodyphus* are a superb model for the study of keystone individuals. Social spiders live in multi-female societies that are inbred and exhibit female-biased sex ratios [33,34] and individuals cooperate in web maintenance, prey capture and alloparental care [35,36]. In *Stegodyphus dumicola* (Araneae, Erasidae), colonies differ substantially in their collective aggressiveness during prey capture [37,38]. Some colonies attack prey rapidly with many attackers, whereas other colonies are slow to attack and do so with few attackers. Such inter-colony variation is a common phenomenon in social insects [39], group-living spiders [38,40], and even vertebrates [41,42], though its causes and ecological consequences are often unknown [43,44]. For social spiders, collective prey capture requires a surprisingly high level of coordination. For instance, evidence suggests that the first few spiders to locate a prey item produce recruitment signals via web-borne vibrations that elicit further attack responses by other individuals [45]. Additionally, social spiders display synchronized pausing behaviour during attacks, where hunting groups alternate bouts of collective approach with collective pauses, which allow colony members to reorient towards struggling prey items in the absence of vibrational interference of other colony members in motion [46]. In *S. dumicola*, colonies’ collective aggressiveness and success are both predicted by the behavioural phenotype of the single boldest individual within the colony. Colonies with just one very bold individual exhibit heightened aggressiveness during prey capture, increased within-colony behavioural variation and gain mass more rapidly in laboratory conditions relative to all-shy colonies [47]. When there are two or more bold individuals within a group, the boldest individual becomes the primary driver of the group's collective behaviour [37]. Recent data suggest that these bold keystones are capable of such influence because they catalyse lasting changes in the boldness/aggressiveness of other group members, such that particularly bold keystones beget bolder behaviour in their normally shy colony mates.

Here we explore what controls the tenure of the keystone individual's long-lasting impacts on group collective behaviour. We term the lingering behavioural changes imposed by the keystone individual ‘legacy effects’. We reason that the longer keystone individuals remain within groups, the greater their influence will be on the behaviour of their fellow group members. Specifically, we test the following hypotheses: (H1) the influence of keystone individuals will linger following their departure but these legacy effects will dissipate with time; (H2) the duration of keystones' legacy effects will scale positively to the amount of time that they spent within the group prior to removal and (H3) keystones with longer tenure will create a larger shift in the behaviour of their fellow group members.

## 2. Material and methods

### (a) Collection and laboratory maintenance

Whole colonies (*N* = 35) of *S. dumicola* were collected in February 2014 along the southern Kalahari Desert near Upington, Northern Cape, South Africa (S 28^°^27′11.8″ E 21^°^22′51.8″). Colonies ranged in size from 232 to 689 individuals. Spiders were transported to the laboratory at the University of Pittsburgh where our experiments were conducted. Only mature females were used in the studies reported here. Colonies were provided a maintenance diet of *ad libitum* domestic crickets twice weekly. All of our experimental colonies were established within three weeks of returning to laboratory as detailed below.

### (b) Boldness assays

We assessed individuals' boldness using an established aversive stimulus assay [38,48]. Trials were initiated by placing each spider in a clean container (radius = 7 cm, depth = 4 cm) and giving them 30 s to acclimate. After 30 s of acclimation, we administered two rapid puffs of air to the anterior prosoma of the spider using an infant nose-cleaning bulb. Like many spiders [49,50], *Stegodyphus* respond to this stimulus by drawing in their legs in the form of a death feign. We then recorded spiders' latency to resume a normal posture and move one complete body length. Trials were terminated after 10 min (600 s). Some individuals resume normal activity rapidly, which we refer to as *bold* behaviour, and others fail to resume normal activity even after 10 min, which we refer to as *shy* behaviour. In *S. dumicola*, individuals' latency to resume movement is highly repeatable over several weeks [37,51,52] and is tightly linked with individuals' tendency to participate in prey capture [48]. Shorter latencies to resume movement correspond to greater boldness, consequently, we subtract individuals' latency from 600 s to generate a more intuitive ‘boldness index’, where larger numbers correspond to greater boldness. All individuals were uniquely tagged using model paint atop their cephalothorax to allow for individual identification.

### (c) Establishing and assaying colonies

Experimental colonies were housed in 490 ml deli containers each containing a tangled ball of poultry wiring to facilitate web construction. Colonies were established 20 days prior to the start of our removal experiment. ‘Keystone removal’ colonies were created with 19 very shy individuals that each exhibited a boldness index score of zero. To this group of 19 spiders, one haphazardly selected, putative keystone individual of variable boldness was added to the group. In some groups, this individual was only slightly bolder than its fellow colony members (e.g. boldness score 5–150) whereas in others it was extraordinarily bold (e.g. boldness scores 400–600). In natural colonies, the vast majority of individuals exhibit a shy behavioural type, thus, the phenotypic ratios used in our study represent a reasonable approximation of those seen in nature [53]. All individuals of an experimental group originated from one natural colony, thus, natural levels of within-group relatedness and familiarity were not adulterated [51,54].

To determine the effect of keystones' tenure on their legacy effects, we established three treatment groups that varied in the duration of exposure to the keystone individual (*N* = 30 colonies per treatment group): (1) a keystone individual was added on the day of colony establishment, i.e. 20 days before its subsequent removal (added on day-20); (2) a keystone was added 10 days after colony establishment (added on day-10) or (3) a keystone was added 15 days after colony establishment (added on day-5). On the same day (day 0), we removed the keystone from all colonies. Thus, colonies harboured their keystone for 20, 10 or 5 days. This procedure held the date of removal and the duration of group tenure constant among treatments. To control for changes to group size, we created paired control colonies containing 18 shy individuals and one putative keystone 20 days prior to the start of the experiment. We then added one very shy individual 20, 10 or 5 days before the start of the experiment (*N* = 30 colonies per control treatment). Then, on the same day that we removed the keystones in the treatment groups, we removed the added shy control individuals from their colonies (see electronic supplementary material, supplement no. 1 for a timeline of the procedure).

Following the removal of the keystone or a control (shy) individual, we assayed the collective aggressiveness during staged prey capture events for all colonies 1, 3, 6, 12 and 24 days after removal. Colony prey capture was assessed by placing a 1.5 × 1.5 cm piece of white paper within the capture web and vibrating it using a handheld vibratory device (GoVibe), producing a controlled and consistent stimulus across all trials. Colonies were not exposed to this vibratory stimulus until day 1 of our experiment (i.e. after the keystones or control individuals had been removed). Thus, the vibrating paper represented a novel prey stimulus for all of our colonies. We recorded the number of attackers that emerged in response to the stimulus over the next 10 min, and noted if the keystone individual was among the attackers. We then provided colonies a four-week-old domestic cricket as a reward for attacking the stimulus. In nature, the number of individuals that respond to prey is positively related to the probability that prey will be successfully subdued [55–57]. This is particularly true for the largest and most profitable prey, which colonies require for their continued survival [58,59].

To determine whether the boldness of the keystone individual influenced the boldness of other colony members, and whether these effects depended on the duration of time that keystones remained within their colony, we conducted further individual boldness assays at the end of our study. After all collective prey capture assays were complete, we dismantled all of the colonies and haphazardly selected three to four shy individuals per colony. Each of these individuals was subjected to three boldness assays as described above, once per day for 3 consecutive days.

### (d) Statistical analyses

To examine whether the effects of the keystone individual dissipate overtime (H1) and whether the duration of the legacy effect varied according to the tenure of the keystone individual (H2), we tested if the number of attackers in the collective prey capture assays changed over time and among treatments using a repeated measures ANOVA. Day of collective prey capture trial, treatment (number of days with keystone or control individual, 5, 10 or 20) and the interaction between day and treatment were effects in the model. Because keystone boldness varied among colonies, and colony aggression scales with the boldness of the keystone individual [47], we further examined the change over time and among treatments in the relationship between the boldness of the keystone individual and number of attackers, which was quantified using Pearson's correlations. We deemed correlations to be significantly different across treatment groups if their 95% CIs failed to overlap, after Laskowski & Pruitt [51]. To examine if shifts in the behaviour of shy group members was influenced by keystones' tenure (H3), we compared the ending boldness indices of spiders among treatments using an ANOVA. We used the average of the three trials of each individual as its boldness index because boldness was highly repeatable (intra-class correlation coefficient (ICC) = 0.42) [60]. We used both colony mean and standard deviation of individual boldness for our comparison among treatments.

Finally, we provide a list of statistics describing the boldness scores of non-keystone group members at the beginning versus the end of our experiments. These values were obtained by calculating the average and standard deviation in boldness scores of all non-keystone individuals at the start of our experiments and comparing these values to the average and standard deviation in boldness scores using three to four representative individuals per colony at the end of our experiments. Statistical analysis was conducted in ‘R’ v. 3.1.2 [61].

## 3. Results

**Figure 1.**
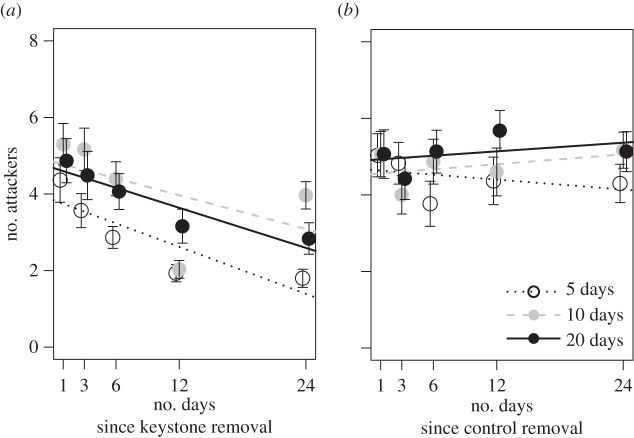
Number of attackers over time. Mean (±s.e.) number of attackers during the five collective prey capture assays of colonies that harboured a bold keystone (*a*) or a control shy (*b*) individual for 5 (white), 10 (grey) or 20 (black) days before removal (*n* = 30 colonies for each treatment). Regression lines are from the repeated measures ANOVA of number of attackers over time.

Regardless of how long the keystone had been in the colony, the number of attackers that responded to prey tended to decrease over time following the removal of the keystone (figure 1*a*). There was a significant change in the number of attackers over time, and overall, the number of attackers was significantly lower in the colonies that contained a keystone for only 5 days (figure 1*a*, repeated measures ANOVA: number of days with keystone (treatment): *F*_2,88_ = 4.904, *p* = 0.0095; test day (time): *F*_4,352_ = 22.336, *p* < 0.0001; time × treatment: *F*_8,352_ = 1.664, *p* = 0.106). No such patterns were noted in control colonies (figure 1*b*, repeated measures ANOVA: number of days with control individual (treatment): *F*_2,87_ = 0.535, *p* = 0.588; test day (time): *F*_4,351_ = 1.314, *p* = 0.264; time × treatment: *F*_8,351_ = 1.342, *p* = 0.222). The data meet the sphericity assumptions according to Mauchly's test, *p* > 0.05, for both control and treatment colonies.

**Figure 2.**
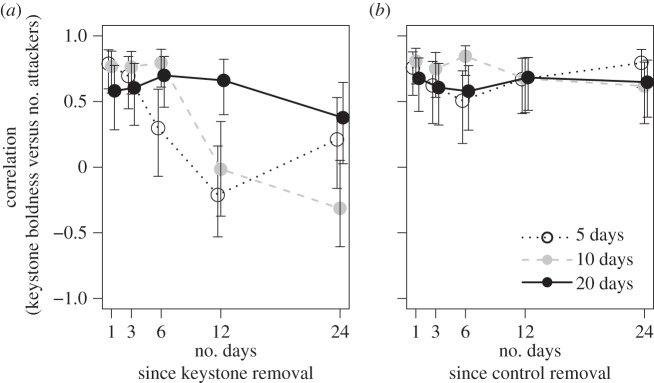
Correlation between keystone boldness index and number of attackers for all trials. Correlation coefficient (±95% CI) of the relationship between the boldness index of the keystone individual and the number of attackers during the five collective prey capture assays of colonies from which a bold keystone (*a*) or a control shy (*b*) individual were removed after 5 (white), 10 (grey) or 20 (black) days (*n* = 30 colonies for each treatment).

As found in previous studies, the boldness of colonies' keystones was tightly associated with the number of spiders that attacked during staged prey capture trials. This association emerged in control colonies regardless of the day that we measured the colony (figure 2*b*; electronic supplementary material, figure S1, Pearson's correlation: all *r* > 0.63, all *p*s < 0.0001). However, for keystone removal colonies, the correlation between the removed keystone's boldness and the aggressiveness of the colony decayed over time. This rate of decay was associated with the duration of time that the keystone had remained in the colony (figure 2*a*). Twelve days after the keystone was removed, the association between the keystone's boldness index and the number of attackers had dissipated in colonies that contained keystones for only 5 or 10 days. However, for colonies that contained a keystone for 20 days prior to its removal, the association between the keystone's boldness and the number of attackers lingered for the entirety of the study, 24 days after the keystone had been removed.

**Figure 3.**
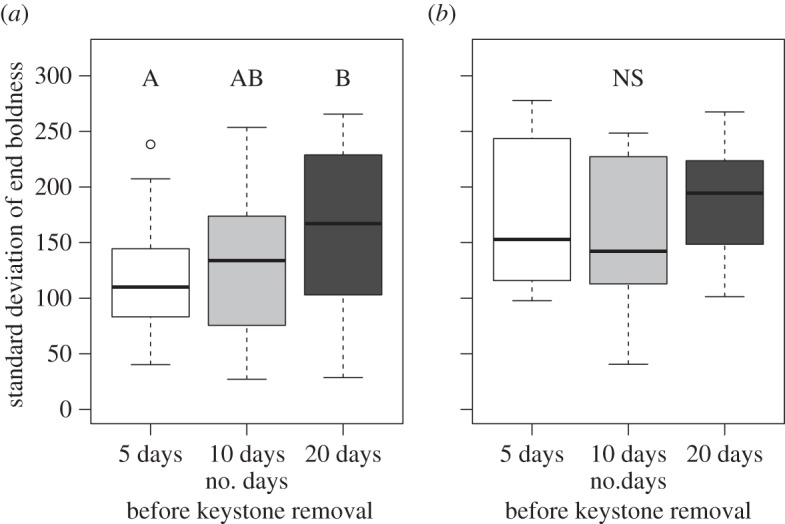
Standard deviation in end boldness score. The standard deviation of boldness index within a colony at the end of the experiment significantly varied among treatments when the keystone individual (*a*) was removed after spending a variable amount of time within the colony (5, 10 or 20 days) prior to removal (*x*-axis), but not when a paired control shy individual (*b*) was removed after spending a variable amount of time within the colony (5, 10 or 20 days).

At the end of our study, inter-individual variation in boldness among formerly shy individuals was highest in colonies that had contained their keystones for longer durations (figure 3*a*, ANOVA: *F*_2,85_ = 3.762, *p* = 0.027). No difference in inter-individual variation was observed among control treatments (figure 3*b*, ANOVA: *F*_2,28_ = 0.688, *p* = 0.511). The colony average boldness index of formerly shy individuals did not differ among keystone removal treatments (ANOVA: *F*_2,86_ = 2.57, *p* = 0.082) or control removal treatments (ANOVA: *F*_2,28_ = 2.286, *p* = 0.12) at the end of experiments. Regardless of treatment, however, the average boldness of formerly shy individuals increased during our experiments. At the beginning of all treatments the mean ± s.d. boldness of the shy individuals was 0 ± 0. At the end of the experiments the average boldness for the various treatments was: keystone removal 5 = 249.63 ± 63.02; keystone removal 10 = 230.41 ± 57.47; keystone removal 20 = 265.87 ± 62.73; control removal 5 = 289.83 ± 31.5; control removal 10 = 253.54 ± 49.01 and control removal 20 = 247.19 ± 59.91.

As seen in previous experiments, keystones' participation in foraging events tapered off rapidly over time in all of our control colonies (electronic supplementary material, figure S2). This is despite the fact that control colonies continued to attack prey with an equally large number of attackers (figure 1*b*). Thus, colony-wide tendency to attack did not decrease, just the tendency for keystones to participate. Notably, although keystones' participation in prey capture diminished over time, the association between the boldness index of the keystone and colonies' collective foraging aggressiveness did not decay (electronic supplementary material, figure S1). Thus, keystone individuals' influence over collective foraging behaviour is not contingent on their consistent participation.

## 4. Discussion

Our understanding of how keystone individuals emerge and operate across the animal kingdom is still in its infancy. In this study, we tested whether the effects of keystone individuals linger following their departure, and whether the duration of keystones' legacy effects scale to the amount of time that they had spent within their groups. As seen in previous studies on *S. dumicola*, bold keystones exhibit a strong influence over the collective foraging behaviour of their society: colonies containing bolder keystones attack prey with many more attackers than other colonies (electronic supplementary material, figure S1). Importantly, this effect does not immediately vanish when the keystone disappears. Instead, the impacts of keystone individuals echo for a variable amount of time following their departure (figures 1 and 2). In other words, keystones exhibit a legacy effect on the collective behaviour of their colony. These data are intriguing because they suggest that social systems have a ‘collective memory’ [10], perhaps mediated through semi-stable changes in individual personalities and/or social network structure, which facilitates system robustness to perturbations that result in the removal of keystone individuals.

The duration of a keystone individual's legacy effects scaled positively to the amount of time that it had spent within its group. First, we found that colonies' collective aggressiveness during prey capture decreased over time following the departure of their keystone, and this decrease was more pronounced in colonies that contained their keystone for less time (20 days versus 5 days; figure 1*a*). Second, we found that the association between keystone individuals' boldness and colony foraging behaviour persisted longer in colonies that contained their keystones for more time (figure 2*a*). These findings suggest that keystones shift the behaviour of their fellow colony members, causing semi-stable changes in collective foraging that persist after the keystone individual's departure. More broadly, these data suggest that keystone individuals (e.g. tutors, leaders) that persist in their roles for longer are more likely to have long-lasting effects on societal behaviour/culture, as seen in human societies and potentially some non-human primates [62,63]. These time-lagged effects of keystone individuals further bare similarity to properties of engineered systems (such as thermostats), physical phenomena (such as rubber elasticity [64]) and some biological processes (such as cell division [65]) in which hysteresis, the dependence of a system's output on its history, reduces the impact of external noise.

Keystone individuals may affect collective behaviour by either performing the task themselves and organizing others to perform it, or by catalysing long-lasting behavioural changes in colony members [30]. Although, recruitment by a few key individuals is common in social insects (e.g. nest site selection in honeybees [66] and foraging in ants [2]), in our system, keystones quickly habituate to novel prey stimuli and rarely participate in prey capture in established colonies (electronic supplementary material, figure S2), even when the stimuli result in prey capture [47]. Yet, despite their lack of participation, their influence over groups' collective behaviour persists (figures 1*b* and 2*b*; electronic supplementary material, figure S1). Thus, keystone effects may be better explained by their catalysis of behavioural variation within their colony (figure 3). Such increased behavioural variation can facilitate efficiency in collective systems [67]. Previous work on *S. dumicola* showed that keystone-induced behavioural diversity persisted when colony members were kept in isolation for months after spending only one week with a keystone individual [47]. Notably, the behavioural metric being catalysed (boldness) is also tightly associated with individuals' tendency to participate in foraging tasks [38,48]. Here we show that even when colonies remain intact after the removal of a keystone individual and group members can interact to assess their social environment [43], changes to individual boldness still persist. Alternatively, or perhaps in addition to these effects, keystone individuals may cause changes to social network structure which, in turn, could change the way the colony behaves *en masse* (N Pinter-Wollman and JN Pruitt 2015, unpublished data). Consistent with this hypothesis, preliminary data suggest that both network structure and within-colony variation in boldness change simultaneously with the presence of a keystone individual. However, the relative contribution of either factor to the phenomena observed here is yet unknown. Similar catalytic mechanisms have been proposed in other kinds of cooperative hunters, like chimpanzees [24,25].

The ecological implications of keystone individuals' ability to enhance colony aggressiveness are substantial. Like wild dogs, social spiders are cooperative hunters that as a group can capture larger and more profitable prey than solitary individuals [55–57]. Subduing large prey becomes especially important as colonies grow larger and web surface area to volume ratio goes down [58]. Because the number of colony members is linearly proportion to web volume, as colonies grow larger, their capture surface area per individual (i.e. foraging potential per individual) goes down, which means less food per individual spider. To overcome this scaling constraint, larger colonies must consistently subdue larger prey with high efficiency [58]. A colony's ability to capture these large prey is directly related to the number of attackers that participate in prey capture [55,56]. Thus, the increased number of attackers associated with the presence of bold individuals in *S. dumicola* is important for the functioning and success of their societies. Indeed, the presence of just one bold colony member increases the collective mass gain of fellow colony members by 200–300% and reduces mortality rates by 40% relative to colonies of all-shy individuals [47]. Fortunately for these societies, the positive influence of keystone individuals on prey capture does not vanish immediately upon their departure.

The effect of keystone individuals on the boldness of other group members scaled with their tenure in the group. All treatments showed an increase in mean boldness of shy individuals from 0 at the start of our experiment to 252.47 ± 59.56 (mean ± s.d.) at the end of the study. However, colonies that contained keystones for longer exhibited a more behaviourally diverse group composition at the end of our study (figure 3) and these behavioural changes were repeatable when individuals were tested multiple times (ICC = 0.42). In colonies that contained their keystones for only 5 days, we saw levels of within-group behavioural variation that were only half of those of colonies that harboured a keystone for 20 days (figure 3*a*). In contrast, the within-group variation of colonies that housed a keystone individual for 20 days before its removal was nearly identical to the behavioural variation observed in the control colonies (figure 3). This finding is impressive because, at the end of the study, keystones had been gone from their colonies longer than they had ever been in them (24 versus 20 days), whereas keystones had never left the control colonies. It is possible that colonies that contained their keystones for only 5 or 10 days never had high within-group variation in boldness; however, this seems unlikely because a previous study demonstrated that just 7 days with a very bold keystone was sufficient to generate a 400% increase in behaviour variation relative to all-shy control groups of *S. dumicola* [47]. Therefore, we deem it more likely that all of our colonies underwent an increase in within-group variation in boldness at some point, but that these effects dissipated following the keystone's disappearance in some treatments. In essence, by polarizing the boldness of other group members, keystones may effectively be creating new (almost) keystones that can replace them when they depart. Granted, other studies on *S. dumicola* [68] and other social *Stegodyphus* [51] have shown that particularly bold group members can emerge spontaneously within groups of all-shy colony members. This process is further associated with enhanced mass gain of the entire social group and increased prey capture and colony defence efficiency (KL Laskowski, PO Montiglio, JN Pruitt 2015, unpublished data). Without a pre-existing keystone, this process takes weeks or months, whereas having a pre-existing keystone individual completes the same process in a matter of days [47]. This further conveys the power of keystone individuals to catalyse important social processes within their colonies.

Our study offers several conceptual and empirical advancements for our understanding of how keystone individuals influence collective behaviour. While most behavioural ecologists are familiar with examples of how innovative behaviours emerge and spread culturally within a population [69–71], our findings differ from such studies in several important ways: (i) we provide evidence that individuals' tendency to become keystone individuals and to initiate legacy effects are associated with their personality type, which is a semi-stable endogenous trait of an individual. While one may reason that the first Japanese macaque to ever wash tubers [69] or wheat [70] was likely to have an innovative behavioural type, there are little data to support such claims. (ii) We provide evidence that keystone individuals are influential because they alter the behavioural tendencies of other individuals in long-lasting and important ways across a variety of situations [47]. In particular, keystone individuals increase the boldness of other colony members and boldness is linked to individuals' tendency to participate in prey capture [53], to assist in web repair/construction [38], and is associated with how individuals respond to predators [37]. This cross-situational influence is in contrast to cultural transmission studies, in which a single specific and highly tangible meme is devised and transmitted [31,70]. (iii) We show that the duration of legacy effects scale to keystone individuals' tenure within groups, which is a question that has never been addressed experimentally in other studies. (iv) Via high levels of replication, we demonstrate that the phenomena identified here are repeatable, robust and can emerge in a variety of social settings (e.g. families and populations). This is in contrast to most published studies on keystone individuals, legacy effects or cultural transmission, which often tend to be descriptive, anecdotal and/or unreplicated. (v) Because of our experimental approach, we are able to link the findings herein with important ecological benefits for colonies, including increased mass gain, survivorship, prey capture efficiency and success during colony defence [47]. Colony success is thought to be the primary driver of individual fitness in social spiders [72]. Finally (vi), the phenomena noted here were observed in social invertebrates, which tend to be underutilized in the field of social learning (but see [73,74]) despite their ecological dominance in terrestrial systems the world over.

## 5. Conclusion

We demonstrate that the legacy effects of keystone individuals have the potential to buffer their societies from radical shifts in collective behaviour associated with their sudden disappearance. However, these effects scale to the former keystone's tenure within the society: groups in which keystones persist for longer periods appear buffered against shifts in collective behaviour, perhaps by producing alternate keystone replacements, whereas societies with short-lived keystones are more likely to exhibit fluctuations in collective behaviour. Although a causal understanding of how keystone individuals exert their influence is still missing for *S. dumicola*, the fact that keystones are able to generate long-lasting shifts in the behavioural tendencies (boldness) of their fellow colony members, even after their departure, highlights the extraordinary influence of keystone individuals on collective outcomes. Few scientists would have presupposed that the behavioural dynamics of social spiders would resemble the hysteresis of physical systems and the collective memory of human societies. The generality of our findings may thus change the kinds of questions asked regarding the interplay between individual variation, social dynamics and collective behaviour.

## Supplementary Material

Figure S1: Relationship between number of attackers and boldness of the keystone individual in control colonies.

## Supplementary Material

Figure S2: Keystone participation in attacks in control colonies.

## Data accessibility

These data are available on Dryad: http://dx.doi.org/10.5061/dryad.31430.
