# Supplementary material for: The legacy effects of keystone individuals on collective behaviour scale to how long they remain within a group
Source: Proc Biol Sci. 2015 Sep 7;282(1814):20151766. doi: 10.1098/rspb.2015.1766 (PMC4571716; doi:10.1098/rspb.2015.1766)
Supplement: Figure S1: Relationship between number of attackers and boldness of the keystone individual in control colonies. [file rspb20151766supp1.doc]

**Media Summary**:

Group composition shapes collective behavior and group success in many animal societies. In some societies, collective behavior is determined by the behavior of just one or a few highly influential individuals, such as leaders or matriarchs, which we term here “*keystone individuals*”. Relying on just one influential individual may be detrimental to group function if the keystone individual dies, unless it leaves a long-lasting legacy of behavioral changes in its group members. We show that as the tenure of a keystone individual in a group increases, so does its long-lasting influence on the behavior of other group members, even after its departure. Uncovering the formation and temporal dynamics of keystone individuals has far-reaching implications for the study of collective behavior.
