# Supplementary material for: The legacy effects of keystone individuals on collective behaviour scale to how long they remain within a group
Source: Proc Biol Sci. 2015 Sep 7;282(1814):20151766. doi: 10.1098/rspb.2015.1766 (PMC4571716; doi:10.1098/rspb.2015.1766)

Figure S1: Relationship between number of attackers and boldness of the keystone individual in control colonies. In each collective prey capture trial of the control colonies (A-E) and in the average of all trials (F) there was a significant ($P < 0.0001$, $r > 0.643$) and positive (slopes > 0.01) relationship between the number of attackers and the boldness index of the keystone individual.

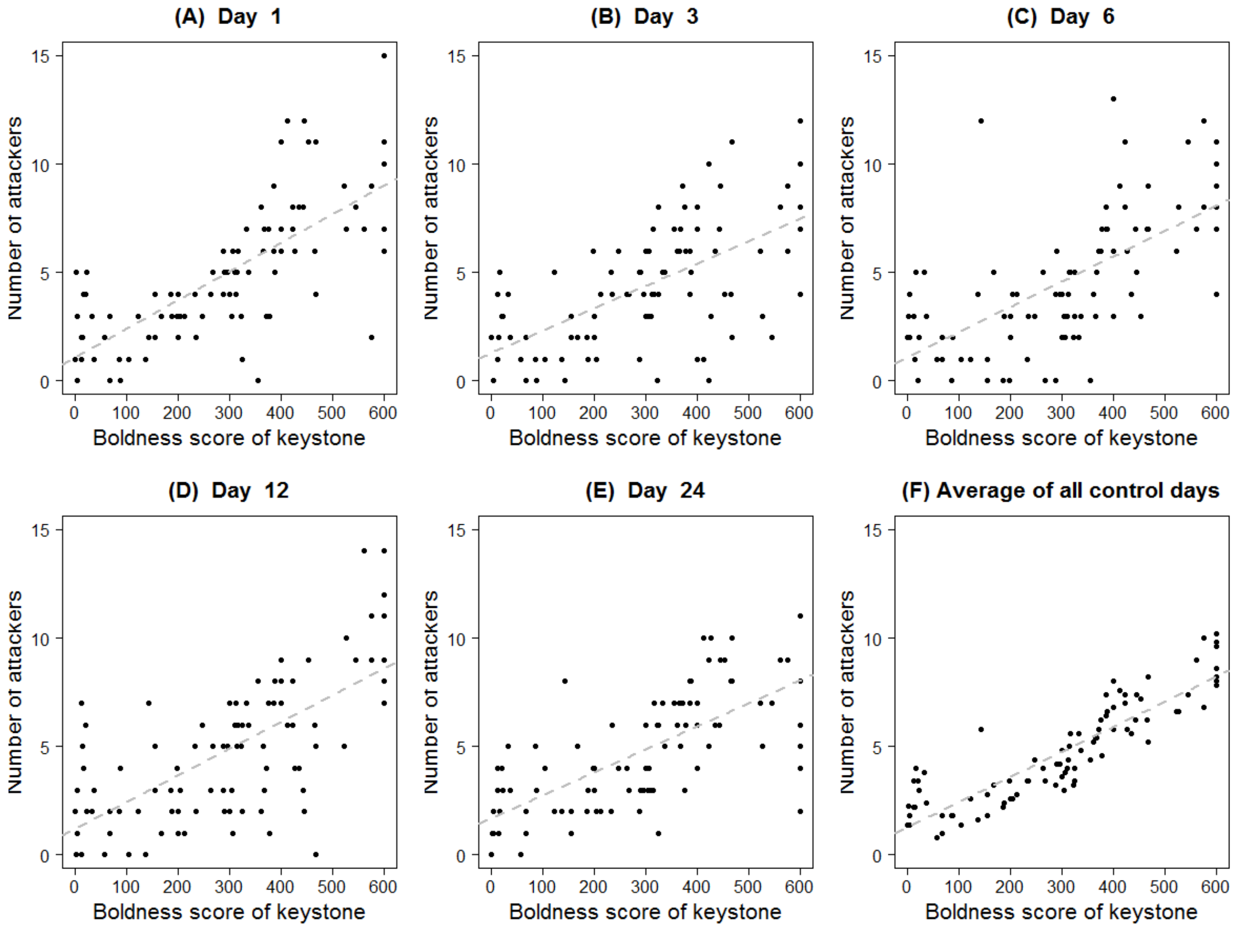

Figure S2: Keystone participation in attacks in control colonies. Proportion of collective prey capture attacks in which the keystone individual participated at each time point when a control shy individual was removed after it had been in the colony for 5 (white), 10 (gray), or 20 (black) days.

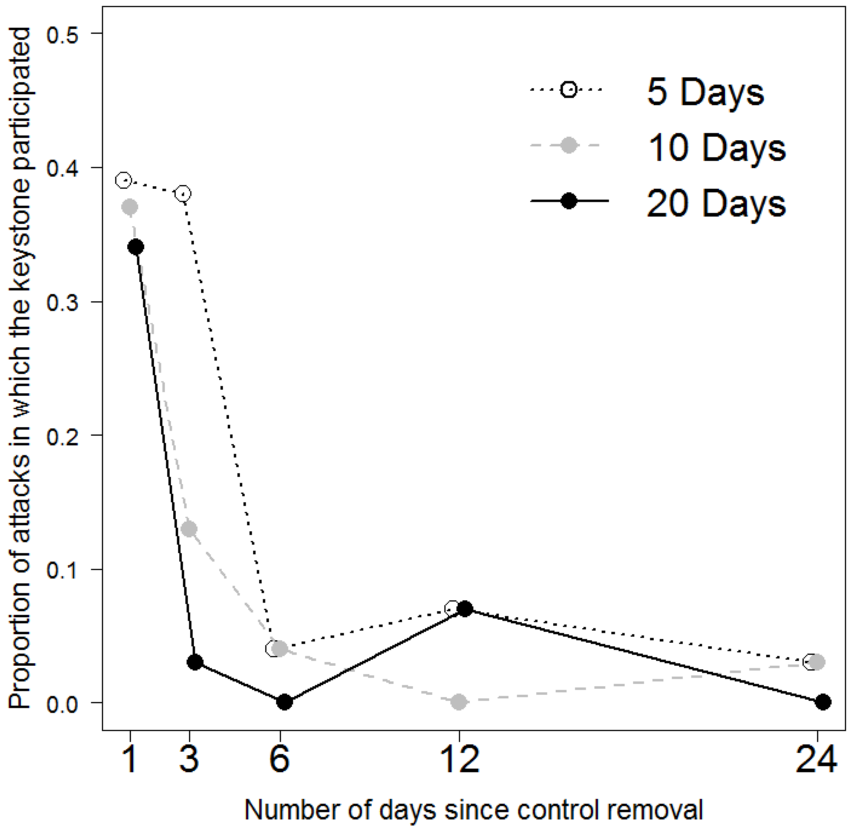

Supplement: Figure S2: Keystone participation in attacks in control colonies. [file rspb20151766supp2.pdf]
